# 

**Published:** 1972-04

**Authors:** 


The
Bristol Medico-Chirurgical Journal
(Incorporating the Medical Journal of the South-West)
Established 1883

Vol. 87 (ii) | APRIL 1972 | No. 322

### BRISTOL MEDICO-CHIRURGICAL JOURNAL

Editor: N. J. Brown, M.B., F.R.C.P., F.R.C.Path.

Published Quarterly

Annual Subscription £2 Post Free

### BRISTOL MEDICO-CHIRURGICAL SOCIETY

**President 1971-72 : Dr. C. C. Morgans**

**President-elect : Dr. James Macrae.**

**Honorary Secretary : Dr. I. S. Bailey, 7 Percival Road, Bristol 8.**

**Honorary Treasurer : Dr. Frank Ross, Bristol Royal Infirmary, Bristol 2.**

The Society was founded in 1874. In 1883 publication of the Journal began and has continued quarterly ever since then. During the years 1949 to 1962 it appeared under the title of "The Medical Journal of the South West".

### THE BRISTOL MEDICO-CHIRURGICAL JOURNAL

**Editorial Committee**

Editor Dr. N. J. Brown, Southmead Hospital, Bristol

Members Dr. Rhys Davies.
Dr. M. B. Lennard.
Professor R. C. Wofinden.
Dr. D. W. Wright.
The Hon. Treasurer and Hon. Secretary of the Society.

Business Manager Dr. P. J. F. Baskett, Frenchay Hospital, Bristol.

Secretary Mr. B. P. Jones, The Library, Medical School, University Walk, Bristol BS8 1TD.

### NOTICE TO CONTRIBUTORS

The Journal is especially concerned to provide a place for the recording of medical thought, interests, and practice in and around Bristol. It therefore welcomes articles that, as well as being of immediate interest to members, will document the local and contemporary medical scene for our successors.

Original articles are invited provided that they have not been published elsewhere. References in the text should be by author's name, with year of publication in parenthesis; they should be listed at the end in alphabetical order, the name of each journal or book being given in full—not abbreviated—and the title of the article added. Illustrations should be supplied ready for reproduction. Line drawing should be in Indian ink on firm white paper or board. The author will be informed if it is found necessary to ask him to pay for the making of blocks.

The following contributions will be especially welcome; notes of forthcoming events, meetings held, degrees conferred, staff appointments, honours and public appointments and other local news.

Twenty-five reprints of his article will be supplied free to each contributor. Quotations for larger quantities will be sent out with proofs and must be ordered when the corrected proofs are returned.

### Bristol Medico-Chirurgical Society

The fourth meeting of the session was held on 12th January, 1972, in the medical school. Dr. D. H. Johnson showed pathological specimens and Dr. J. Roylance demonstrated skiagrams. The subject of "Accidents in the Home" was discussed by Mr. C. P. de Fonseka, Epidemiologist, and Mr. John Roberts, Health Economist.

On 9th February Dr. W. Lumsden Walker read a paper on "Crises of Adolescence" which is published elsewhere in this number of the Journal: Dr. M. E. H. Halford showed pathological specimens and Dr. R. J. Burwood demonstrated skiagrams.

At both meetings Mr. B. P. Jones, medical librarian, arranged his usual interesting display of medical books.

---

### EXAMINATION RESULTS AND DISSERTATIONS APPROVED

### University of Bristol

### FACULTY OF MEDICINE

**M.D.** Ralph Ernest BARRY
Anthony Arthur CODD
Roger SNOOK

**Ph.D.** Abdul-Kadir Jassim AL-SHAIKHLY
Moses Joseph BOJRAB
Mary KEAR
Frances Mary PONSFORD
Jennifer Anne REES
Alan Stuart ROBINSON

**M.B., Ch.B.** Edgar Munir ABBAS
Susan ACKERMAN
Maureen Gwendoline BEACHAM
Catherine Wray BERRY
William John BURROWS
David John Robert CANNELL
Christopher John CUTHBERT
Elizabeth Ann EARLAND
Michael James John GRAY
Patrick Charles HALSE
Stuart HANKEY
Martin Ross HARRISON
Gail Margaret HARVEY
Richard David HUTTON
Barbara Anne KING
Ka-Kon KWAN
Patricia Mary LOOSMORE
William Peter MASON
Christopher John MEW
David Alun Lloyd MORGAN
Andrew Paul NASH
Timothy Savile PARDOE
Eric Richard Edward THOMSON
Elizabeth Mary WALKER
Peter Hendy WILKINS

